# Human Umbilical Cord Mesenchymal Stem Cell-Derived Extracellular Vesicles Promote the Proliferation of Schwann Cells by Regulating the PI3K/AKT Signaling Pathway via Transferring miR-21

**DOI:** 10.1155/2021/1496101

**Published:** 2021-09-11

**Authors:** Yongbin Ma, Dan Zhou, Huanyan Zhang, Liming Tang, Fen Qian, Jianhua Su

**Affiliations:** ^1^Department of Central Laboratory, Jintan Hospital, Jiangsu University, 500 Avenue Jintan, Jintan 213200, China; ^2^Department of Pain, Zhi'xi Town Center Hospital, 128 Zhenxing South Road, Jintan 213251, China

## Abstract

As an alternative mesenchymal stem cell- (MSC-) based therapy, MSC-derived extracellular vesicles (EVs) have shown promise in the field of regenerative medicine. We previously found that human umbilical cord mesenchymal stem cell-derived EVs (hUCMSC-EVs) improved functional recovery and nerve regeneration in a rat model of sciatic nerve transection. However, the underlying mechanisms are poorly understood. Here, we demonstrated for the first time that hUCMSC-EVs promoted the proliferation of Schwann cells by activating the PI3K/AKT signaling pathway. Furthermore, we showed that hUCMSC-EVs mediated Schwann cell proliferation via transfer of miR-21. Our findings highlight a novel mechanism of hUCMSC-EVs in treating peripheral nerve injury and suggest that hUCMSC-EVs may be an attractive option for clinical application in the treatment of peripheral nerve injury.

## 1. Introduction

Peripheral nerve injury has become the pivotal issue in human health because of their higher prevalence [1, 2]. These injuries often cause motor and sensory dysfunction, even permanent disability. Although a large number of surgical procedures have been performed to repair peripheral nerve injuries, the clinical outcome is still unsatisfactory [3]. Therefore, the development of new therapeutic strategies to improve peripheral nerve regeneration and repair is of great importance.

Schwann cells play a key role in peripheral nerve regeneration. Schwann cells are glial cells in the peripheral nervous system, enclose neuronal axons to form myelin sheaths, and are essential for maintaining axonal survival and integrity [4, 5]. Following nerve injury, Schwann cells are reprogrammed into repair phenotypes to provide biochemical signals and spatial cues, which support neuronal survival, axon regeneration, and redominance of target organs [6, 7]. Given that their pivotal role in the repair of peripheral nerve injury, regulating the biological function of Schwann cells may be an effective strategy to accelerate peripheral nerve regeneration and repair.

In recent years, studies have shown that mesenchymal stem cell- (MSC-) based therapy is considered to be a novel approach for peripheral nerve injury because they not only significantly promote axonal regeneration but also elevate the recovery of motor function [8]. It is well known that bioactive compositions secreted by paracrine have been identified as a key mechanism of action of MSCs [9, 10]. Extracellular vesicles (EVs), nanosized (50-200 nm) vesicles with a lipid bilayer membrane, released by almost all cell types, are a new mechanism for communication between cells [11]. More specifically, donor cell-derived EVs can mediate the biological function of recipient cells by transferring proteins and functional genetic material such as RNA [12, 13]. Notably, emerging evidence suggests that transplantation of MSCs or MSC-EVs exhibits similar therapeutic effects in promoting nerve regeneration and improving motor function recovery after peripheral nerve injury [14, 15]. Moreover, the application of MSC-EVs was proved to be safer than MSC administration, which could avoid some inherent risks, including microcirculatory obstruction, arrhythmia, cellular immune response, and carcinogenic mutation [16, 17]. Obviously, MSC-EVs represent a new cell-free therapy alternative to MSCs in the treatment of peripheral nerve injury.

Previous data in our laboratory have demonstrated that intravenous injection of human umbilical cord mesenchymal stem cell- (hUCMSC-) derived EVs significantly promoted nerve regeneration and motor function recovery in a rat sciatic nerve transection model [18]. However, the underlying mechanism is still unclear. In this study, we further attempted to determine the relevant mechanism of hUCMSC-EV effectiveness, especially on the biological function of Schwann cells.

## 2. Materials and Methods

### 2.1. Isolation and Characterization of hUCMSCs

Fresh umbilical cord samples were obtained from consenting mothers at Jintan Hospital affiliated of Jiangsu University (Jintan, China) with the approval of the ethics committee of Jintan Hospital (ethical approval number: KY-2019001). hUCMSCs were extracted from a fresh umbilical cord according to the previously published method [18, 19]. In brief, the umbilical cord was washed 2-3 times with phosphate-buffered solution (PBS) containing penicillin and streptomycin (pen/strep; Gibco, Carlsbad, CA), and umbilical cord blood vessels were carefully removed. The remaining tissue was subsequently cut into 1 mm^3^-sized sections with scissors that were individually attached to the substrate of culture plates and maintained in umbilical cord stem cell culture medium (Cyagen, Guangzhou, China) at 37°C in a 5% CO_2_ incubator. After the initial culture, the medium was changed every 3 days until the well-developed colonies of spindle-like cells appeared about 10 days later. The cells were then digested with 0.25% trypsin-EDTA (Beyotime, Nantong, China) and passaged into new flasks for further expansion. The human umbilical cord MSCs (hUCMSCs) from passages 3-7 were used in all the next experiments.

The adipogenic and osteogenic differentiation ability of hUCMSCs was identified by Oil Red O and alkaline phosphatase staining as previously described [18, 20]. Briefly, hUCMSCs from passage 3 were seeded into 6-well plates and cultured with OriCell™ hUCMSC adipogenic differentiation or osteogenic differentiation medium (Cyagen) as described by the manufacturer. Following 14 days of adipogenic differentiation, the cells were stained with Oil Red O staining kit (Beyotime) according to the manufacturer's instructions. Adipogenic differentiation was demonstrated by the intracellular accumulation of red lipid droplets. 21 days after osteogenic differentiation, the cells were stained with alkaline phosphatase detection kit (Beyotime) according to the manufacturer's instructions. Blue-purple bodies were identified as alkaline phosphatase positive.

The typical surface markers of P3 passage hUCMSCs were detected by flow cytometry (BD Accuri C6 flow cytometer; BD Biosciences, San Jose, CA, USA) as previously described [21]. Fluorescein isothiocyanate- (FITC-) conjugated or phycoerythrin- (PE-) conjugated monoclonal antibodies specific for CD19, CD29, CD90, and CD105 were purchased from BD Biosciences. Identical concentrations of FITC or PE mouse nonimmune isotypic IgG were used as negative controls (BD Biosciences).

### 2.2. Cell Culture

Schwann cells are the principal glial cells in the peripheral nervous system. The rat Schwann cell line RSC96, purchased from the Cell Bank of Chinese Academy of Sciences, has been widely used as a cell line model for this cell type [22, 23]. RSC96 cells were cultured with a high-glucose DMEM supplemented with 10% fetal bovine serum (FBS) at 37°C in a 5% CO_2_ incubator. In order to eliminate the interference of EVs in FBS, EV-free medium was used in related experiments involving hUCMSC-EVs.

### 2.3. Isolation and Identification of hUCMSC-EVs

hUCMSCs were seeded in100 mm dish at a density of 2 × 10^6^ cells, cultured until 80% confluent, washed twice with phosphate-buffered solution (PBS), and reincubated with serum-free medium with a volume of 6 mL for 24 h. The supernatant of hUCMSCs was collected, followed by hUCMSC-EV isolation through ultracentrifugation as previously described [19]. Briefly, the hUCMSC culture supernatant was centrifuged at 300 × *g* for 5 min at room temperature and 2000 × *g* for 30 min at 4°C to remove cell and cell debris. And then, the hUCMSC supernatant continued to be centrifuged at 100,000 × *g* for 90 min at 4°C. After that, the pellets were gathered and resuspended in sterile PBS, followed by repeat centrifugation once at 100,000 × *g* for 90 min at 4°C to isolate hUCMSC-EVs.

The protein content of the concentrated hUCMSC-EVs was detected using BCA protein assay kit (Beyotime, Nantong, China) following the manufacturer's instructions. Then, hUCMSC-EVs were resuspended in PBS, aliquoted in EP tube, and stored at -80°C.

The morphology of hUCMSC-EVs was detected using a transmission electron microscope (TEM) (JEM-1200EX; JEOL Ltd., Tokyo, Japan) as previously described [24]. The particle size distribution of hUCMSC-EVs was determined by nanoparticle trafficking analysis (NTA) using a NanoSight NS300 system (Malvern Instruments Ltd., Worcestershire, UK).

### 2.4. Cell Transfection

hUCMSCs at 60% confluency were transfected with a concentration of 100 nM miR-21 inhibitor or negative control (inhibitor control) by using Lipofectamine 2000 (Invitrogen, Carlsbad, CA) in Opti-MEM (Invitrogen) according to the manufacturer's instructions. Then, the EVs were extracted according to the above protocol described and were named as miR-21 inhibitor-EVs and NC-EVs, respectively. The synthetic miR-21 inhibitor and negative control were purchased from GenePharma (Shanghai, China).

### 2.5. Animal Model

All animal experimental procedures and protocols were reviewed and approved by the Animal Investigation Ethics Committee of Jiangsu University (Permit Number: SYXK [Su] 2018-0053) and were performed in accordance with the Guidelines for the Care and Use of Laboratory Animals from the National Institutes of Health.

Adult male Sprague-Dawley (SD) rats (weighing: 220-230 g) were used to establish rat sciatic nerve transection injury model according to our previously published method [18]. Specifically, the left sciatic nerve was fully exposed in a sterile environment after rat anesthesia, and a 5 mm long gap at 1 cm above the sciatic nerve bifurcation was created. And then, the proximal and distal nerve stump (1 mm) was inserted into both ends of the silicone tube (7 mm) and fixed with a 10-0 nylon suture. A 5 mm long gap was maintained. After 24 h, the model was randomly assigned to two groups (*n* = 6 per group): hUCMSC-EV group and control group. The final amount of hUCMSC-EVs used for in vivo animal study was 100 *μ*g/200 *μ*L PBS for each animal (*n* = 6). Equal volume of PBS (200 *μ*L) was used for the control group (*n* = 6). The hUCMSC-EV group or the control group was injected via tail vein using a precooled microinjector (Shanghai Medical Laser Instrument, China). Rats were euthanized and nerve conduits were harvested at 57 days.

To explore the effectiveness mechanism of hUCMSC-EVs, another 12 rats were randomly divided into two groups (*n* = 6 per group), and 100 *μ*g proteins of miR-21 inhibitor-EVs or NC-EVs were injected into rat sciatic nerve transection model through the tail vein. Rats were euthanized and nerve conduits were harvested at 57 days.

### 2.6. Tracing hUCMSC-EVs In Vitro and In Vivo

For tracing *in vivo*, hUCMSC-EVs were labeled with DiR (Invitrogen, Carlsbad, CA, USA) according to our previously described method [18]. DiR-labeled EVs or PBS (PBS served as control) were injected into rat sciatic nerve transection model through the tail vein. After injection for 24 h, rats were euthanized and nerve conduits were harvested.

For tracing *in vitro*, hUCMSC-EVs were labeled using the CM-Dil (Invitrogen) following the manufacturer's instructions. Then, CM-Dil-labeled EVs were reextracted and incubated with RSC96 cells for 4 h on coverslips in a 24-well plate. Following fixing with 4% paraformaldehyde and staining with DAPI (Servicebio, Wuhan, China), the cells were viewed under a Nikon Eclipse 80i fluorescence microscope.

### 2.7. Immunofluorescence Staining

Immunofluorescence staining was performed according to the previously published method [18]. In brief, the harvested nerve conduits were embedded in paraffin fixation. After that, the paraffin-embedded sections were blocked and labeled with rabbit anti-rat S100 (1 : 100 dilution, Servicebio) and mouse anti-BrdU (1 : 100 dilution, Servicebio). Alexa Fluor 488 goat anti-rabbit antibody (1 : 400 dilution, Servicebio) or Cy3 goat anti-mouse antibody (1 : 300 dilution, Servicebio) was used as the secondary antibody. DAPI was used to stain the nucleus.

### 2.8. Cell Viability and Proliferation Assay

RSC96 cells (5 × 10^3^) in 96-well plates were treated with hUCMSC-EVs (medium served as control) or miR-21 inhibitor-EVs (inhibitor NC-EVs served as control) for 48 h. Cell viability was determined using cell counting kit-8 (Biosharp, Beijing, China), according to the manufacturer's manuals.

Colony-forming assay was performed to detect the proliferation of cells. RSC96 cells (5 × 10^2^) in 35 × 10 mm dish were treated with hUCMSC-EVs (medium served as control) or miR-21 inhibitor-EVs (inhibitor NC-EVs served as control) for 10 days. Then, the cells were fixed with 4% formaldehyde and stained with 0.1% crystal violet followed by colony counting.

### 2.9. Western Blotting

Protein samples from hUCMSC-EVs, supernatant, and RSC96 cells treated with hUCMSC-EVs or miR-21 inhibitor-EVs were extracted and quantified for Western blot analysis in accordance with our previously described procedure [24]. The monoclonal antibody of AKT (1 : 1000, CST), phospho-AKT (1 : 1000 dilution, CST), PI3K (1 : 1000, CST), phospho-PI3K (1 : 1000 dilution, Affinity Biosciences), and *β*-actin (1 : 1000 dilution, CST) was used as the primary antibodies. HRP-linked anti-rabbit IgG (1 : 2000 dilution, CST) was used as the second antibody, and *β*-actin was used as the internal control.

### 2.10. qRT-PCR Analysis

Total RNA was extracted from RSC96 cells treated with hUCMSC-EVs or miR-21 inhibitor-EVs. cDNA was synthesized using All-in-One™ First-Strand cDNA Synthesis Kit (Genecopoeia, Germantown, MD) according to the manufacturer's protocols. Then, qRT-PCR analysis was performed with All-in-One™ qPCR Mix (Genecopoeia) and the primers purchased from Genecopoeia in an ABI system (Applied Biosystems, Foster City, CA, USA), following manufacturer's protocol. The relative expression of miRNA was normalized to U48 and determined by the 2^–*ΔΔ*Ct^ method.

### 2.11. Statistical Analysis

All data are presented as the mean ± standard error of the mean. Statistical analysis of data was performed using the GraphPad Prism software (Version 5.0; La Jolla, CA). The comparison between the two groups was assessed by Student's *t-*test. *P* value less than 0.05 was regarded as statistical significance.

## 3. Results

### 3.1. Identification of hUCMSCs and hUCMSC-EVs

**Figure 1:**
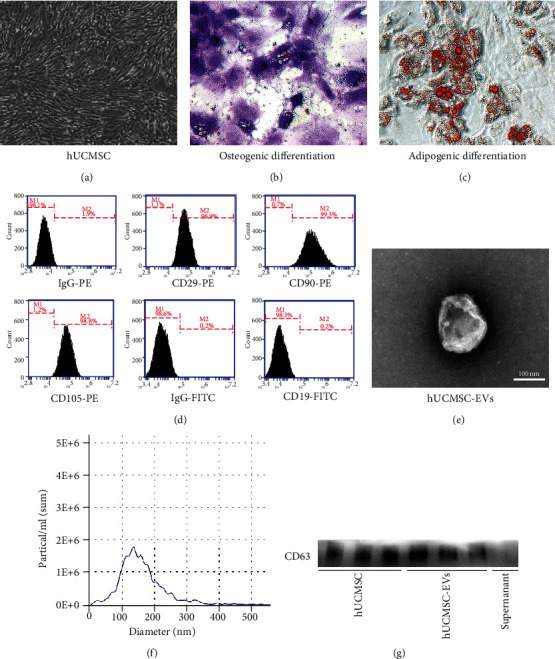
Characterization of hUCMSCs and hUCMSC-EVs. (a) Morphology of passage 3 hUCMSCs under white light microscope, magnification: ×40. (b, c) Induction of osteogenic (alkaline phosphatase stained) and adipogenic differentiation (Oil Red O stained) of hUCMSCs, magnification: ×200. (d) Phenotypic markers related to hUCMSCs by flow cytometry analysis. (e) Representative images of hUCMSC-EVs using a transmission electron microscope (TEM), scale bar: 100 nm. (f) The particle size distribution and concentration of hUCMSC-EVs by nanoparticle tracking analysis (NTA). (g) Western blot analysis of CD63 expression in hUCMSC-EVs and hUCMSCs. Supernatant obtained during EV isolation by ultracentrifugation was regarded as negative control.

Based on the minimal identification criteria standard of human MSC by the International Society of Cell Therapy [25], herein, our results showed that cells derived from the human umbilical cord have a spindly and fibroblast-like morphology (Figure 1(a)). Furthermore, these cells exhibited the ability to differentiate into osteogenesis and adipogenesis (Figures 1(b) and 1(c)). More importantly, the immunophenotype of these cells was detected by flow cytometry, suggesting that they were positive for CD29, CD90, and CD105, but negative for CD19 (Figure 1(d)). All of these results suggest that we had successfully produced hUCMSCs.

In addition, we further purified and identified hUCMSC-EVs. hUCMSC-EVs were cup-shaped with double-layer membrane structure; the average particle size of hUCMSC-EVs was about 141.2 nm with a size distribution of 30 to 450 nm (Figures 1(e) and 1(f)). Moreover, we further confirmed that the level of CD63 was enriched in hUCMSC-EVs and hUCMSCs, while almost no expression was observed in the supernatant (Figure 1(g)). These results indicate that we had efficiently generated hUCMSC-EVs, as confirmed on the basis of the criteria defined by the International Society for Extracellular Vesicles [26].

### 3.2. hUCMSC-EVs Promote Nerve Regeneration

**Figure 2:**
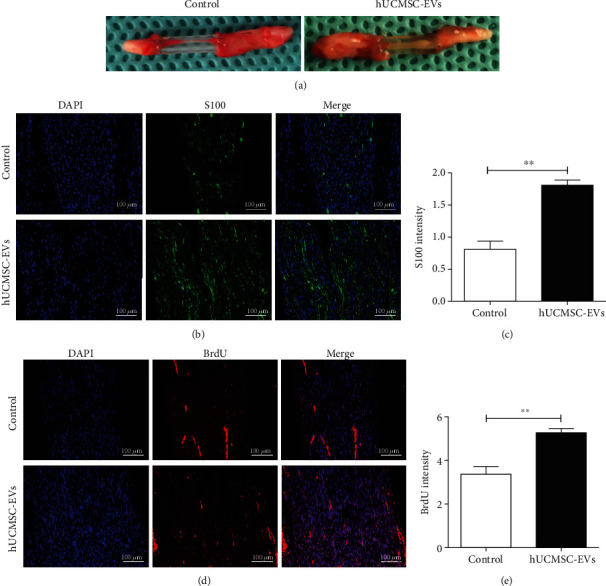
hUCMSC-EVs promoted the proliferation of Schwann cells *in vivo*. (a) Representative picture showing the macroscopic appearance of regenerated nerve in nerve conduit after sciatic nerve transection at 57 days. (b, c) S100 immunofluorescence staining (green) and statistical analysis of S100 intensity, scale bar: 100 *μ*m. (d, e) BrdU immunofluorescence staining (red) and statistical analysis of BrdU intensity, scale bar: 100 *μ*m. Data are expressed as the mean ± SEM, ^∗∗^*P* < 0.01*vs.* control (PBS group).

To clarify the beneficial effects of hUCMSC-EVs, we used a rat model of sciatic nerve transection. After 57 days of administration, the regenerated nerve in silicone tube was obviously thick compared with that in the control group (Figure 2(a)). Schwann cells are the main glial cells in the peripheral nervous system and play a key role in peripheral nerve regeneration [6, 7]. We next examined the Schwann cell proliferation via fluorescence staining. S100 is a specific marker for proliferative Schwann cells [27]. S100 is a major extrinsic membrane protein secreted by Schwann cells [28]. The result showed that the hUCMSC-EVs as well as the control group had positive expression of S100, while the hUCMSC-EV group showed stronger expression of S100 (Figures 2(b) and 2(c)). Similarly, BrdU fluorescence staining further showed that hUCMSC-EVs promoted the proliferation of Schwann cells (Figures 2(d) and 2(e)). Taken together, these findings suggest that hUCMSC-EVs promote Schwann cell proliferation.

### 3.3. hUCMSC-EVs Promote Schwann Cell Proliferation via Activating the PI3K/AKT Signaling Pathway

**Figure 3:**
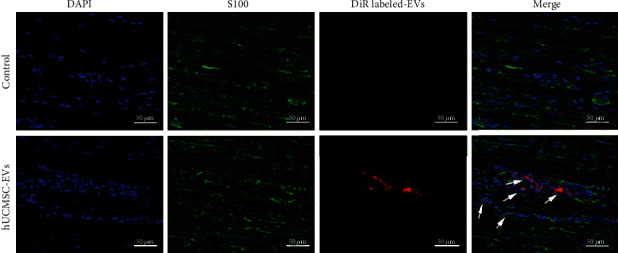
Uptake of hUCMSC-EVs by nerve stump tissue. After tail vein administration, fluorescence staining confirmed the location of DiR-labeled hUCMSC-EVs in Schwann cells, scale bar: 50 *μ*m.

**Figure 4:**
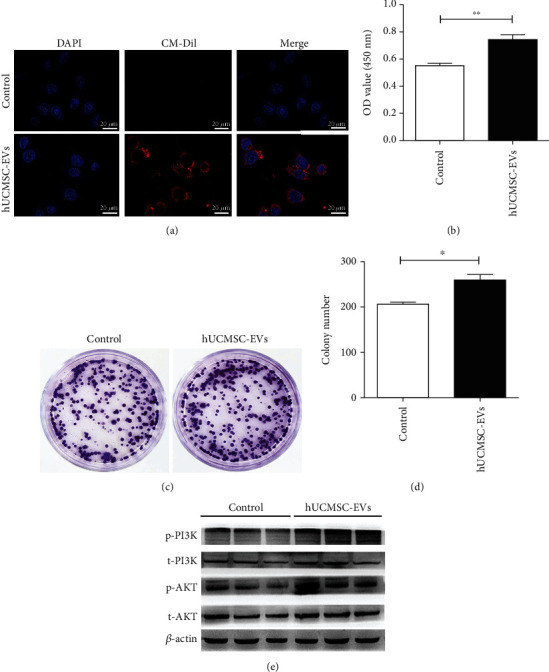
hUCMSC-EVs promoted the proliferation of Schwann cells *in vitro*. (a) CM-Dil-labeled hUCMSC-EVs are internalized by RSC96 cells, scale bar: 20 *μ*m. (b) The proliferation activity of RSC96 cells by CCK-8 assay. (c, d) The proliferating ability of RSC96 cells by colony-forming assay. Data are expressed as the mean ± SEM, ^∗^*P* < 0.05, ^∗∗^*P* < 0.01*vs.* control (medium without EVs). (e) Western blot analysis of the protein expression of p-PI3K, PI3K, p-AKT, and AKT in Schwann cells.

Next, we evaluated whether hUCMSC-EVs were internalized. In an *in vivo* study, DiR-labeled hUCMSC-EVs were present at the site of sciatic nerve injury. Fluorescence staining results confirmed that hUCMSC-EVs gathered around the nuclei of Schwann cells (Figure 3), demonstrating that Schwann cells may be the receptor cells of hUCMSC-EVs. Of note, *in vitro* studies showed that hUCMSC-EVs surrounded the nuclei and lined the inner surface of RSC96 cells (Figure 4(a)). These results provided strong evidence that Schwann cells effectively internalize hUCMSC-EVs *in vivo* and *in vitro*.

As a medium of intercellular communication, EVs contribute to donor cell-mediated biological effects [11]. To confirm the modulatory role of hUCMSC-EVs, we examined the effect of hUCMSC-EVs on Schwann cell proliferation. The results of CCK-8 and colony-forming assay showed that hUCMSC-EVs significantly increased the proliferation ability of RSC96 cells (Figures 4(b)–4(d)). PI3K/AKT is a common signaling pathway that regulates cell proliferation [29]. Activation of the PI3K/AKT signaling pathway correlates with Schwann cell proliferation [29, 30]. Here, we tested the expression levels of p-AKT, AKT, p-PI3K, and PI3K by Western blot analysis. As shown in Figure 4(e), hUCMSC-EVs remarkably increased the expression levels of p-AKT and p-PI3K in RSC96 cells. Taken together, these results indicate that hUCMSC-EVs promote Schwann cell proliferation by activation of the PI3K/AKT signaling pathway.

### 3.4. miR-21 Is Critical for hUCMSC-EV-Mediated Proliferation of Schwann Cells

**Figure 5:**
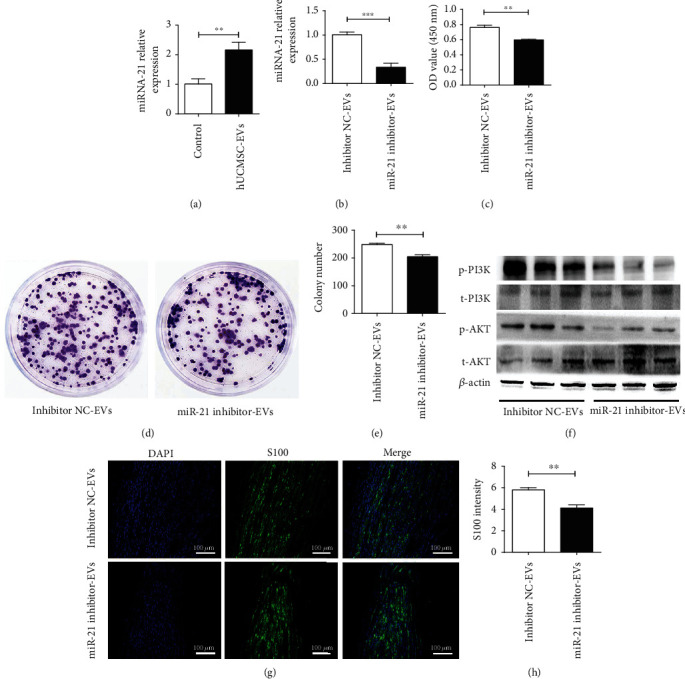
miR-21 is critical for hUCMSC-EV-mediated proliferation of Schwann cells. (a, b) RT-PCR analysis of relative expression of miR-21 in RSC96 cells treated with hUCMSC-EVs or miR-21 inhibitor-EVs. (c–e) The effects of miR-21 inhibitor-EVs on RSC96 cell proliferation as determined by CCK-8 assay and colony-forming assay, respectively. (f) The effects of the protein expression of p-PI3K, PI3K, p-AKT, and AKT in RSC96 cells treated with miR-21 inhibitor-EVs or inhibitor NC-EVs. (g, h) The expression intensity of S100 in regenerated nerve tissue after injection of miR-21 inhibitor-EVs or inhibitor NC-EVs. Data are expressed as the mean ± SEM, ^∗∗^*P* < 0.01 and ^∗∗∗^*P* < 0.001 for hUCMSC-EVs *vs.* control (medium without EVs) or miR-21 inhibitor-EVs *vs.* inhibitor NC-EVs.

A recent study showed that miR-21 plays an important role in promoting Schwann cell proliferation during injured peripheral nerve repair [23]. Dramatically, we found that hUCMSC-EVs enriched high levels of miR-21 (Figure 5(a)). In addition, the expression of miR-21 in RSC96 cells treated with hUCMSC-EVs was significantly higher than that in the control group, indicating that hUCMSC-EV-mediated upregulation of miR-21 in RSC96 cells can be attributed to direct transfer of miR-21. To further clarify whether miR-21 is the key to the beneficial effect of hUCMSC-EVs on Schwann cell proliferation, we knocked down the expression level of miR-21 in hUCMSC-EVs using miR-21 inhibitor (named miR-21 inhibitor-EVs) and administered negative control (named NC-EVs) (Figure 5(b)). Then, miR-21 inhibitor-EVs or their control inhibitor NC-EVs were administered to RSC96 cells. Our results showed that miR-21 inhibitor-EVs significantly reduced the proliferation of RSC96 cells compared with the inhibitor NC-EVs, as determined by CCK-8 and colony-forming assay (Figures 5(c)-5(e)). Western blot analysis showed that miR-21 inhibitor-EVs significantly decreased the protein expression levels of p-AKT and p-PI3K in RSC96 cells (Figure 5(f)). Moreover, miR-21 inhibitor-EVs or inhibitor NC-EVs were also injected into rat sciatic nerve transection model. Compared with the inhibitor NC-EVs, miR-21 inhibitor-EV treatment strongly inhibited the positive expression of S100 (Figures 5(g) and 5(h)). Taken together, these findings indicate that miR-21 silencing reduces hUCMSC-EV-mediated proliferation of Schwann cells.

## 4. Discussion

The development of cell-free therapeutics based on the use of MSC-EVs as an alternative MSC-based therapy has already shown promise in regenerative medicine [31]. In this study, we further characterized the hUCMSC-EVs, and we showed that hUCMSC-EVs contribute to sciatic nerve regeneration by promoting Schwann cell proliferation.

MSCs from the umbilical cord are convenient to harvest in a noninvasive manner and have greater amplification capability, lower immunogenicity, and fewer minimal ethical concerns than other adult counterparts [32]. Apart from this, hUCMSCs are also considered as a better choice for clinical application and hUCMSC cell bank has been established in many countries [33]. Accumulating evidence from studies has demonstrated that hUCMSCs have greater paracrine effects and can potentiate peripheral nerve axonal regeneration and functional recovery through these effects [32]. As described in the review [34, 35], most of these effects are mediated by their secretory EVs. Thus, we chose hUCMSCs as the source of stem cell-derived EVs and tested the therapeutic potential of hUCMSC-EVs for peripheral nerve injury using a rat model of sciatic nerve transection.

Schwann cells are functional cells for peripheral nerve regeneration [7]. Although we could not rule out the possibility that hUCMSC-EVs directly act on neuron axons or the indirect effect target on the other cells (such as macrophages), we found for the first time that intravenous injection of hUCMSC-EVs aggregated to the site of sciatic nerve injury and be uptaken by Schwann cells, indicating that Schwann cells were the effector target cells of hUCMSC-EVs. Consistent with a previous study [15, 36], hUCMSC-EVs also significantly promoted the proliferation of RSC96 cells by activating the PI3K/AKT signaling pathways. Our research results further support the concepts of biological effects of EVs as mediators of cell-to-cell communication [37]. By contrast, Zhou et al. [38] reported that bone marrow mesenchymal stem cell-derived EVs inhibited the proliferation of Schwann cells and promoted their apoptosis. The difference of these results may be due to different MSC sources and different culture conditions.

miR-21, as one of the most deeply studied miRNAs, had been reported to participate in peripheral nerve injury and repair [23]. This evidence points that miR-21 promoted Schwann cell proliferation [23]. In addition, miR-21 also inhibited Schwann cell apoptosis induced by oxidative stress [39]. Numerous studies have confirmed that MSC-EVs modulate the biological activity of target cells by transferring specific miRNAs [40–42]. Interestingly, the abundance of miR-21 was relatively higher in hUCMSC in accordance with previous high-throughput sequencing results [40]. Therefore, we hypothesized that the beneficial effect of hUCMSC-EVs may be through the delivery of miR-21. Indeed, we observed that the expression of miR-21 was significantly increased after hUCMSC-EVs treated RSC96 cells, while the proliferation of RSC96 cells significantly reduced and inhibited the activation of the PI3K/AKT signaling pathway related to RSC96 cell proliferation after knocking down the expression of miR-21 in hUCMSC-EVs. In addition, an in vivo experiment has further revealed that hUCMSC-EVs knock down the expression of miR-21 and inhibit the proliferation of Schwann cells. These positive results support our hypothesis. Our data show that miR-21 is the key modulator of hUCMSC-EVs mediating Schwann cell proliferation. However, the contents of hUCMSC-EVs are heterogeneous; we cannot rule out the possibility that other miRNA, proteins, or lipids have beneficial effects on Schwann cells. As reported by Zhang et al. [43], EVs derived from human embryonic neural stem cells could inhibit cardiomyocyte apoptosis by transporting HSP-70, activating AKT and mTOR signaling pathways. These interesting possibilities await further investigation in our future studies.

## 5. Conclusion

In conclusion, our study demonstrates that the therapeutic effect of hUCMSC-EVs on sciatic nerve injury is mediated at least partly via transferring miR-21. miR-21 transferred into Schwann cells by hUCMSC-EVs promotes the proliferation of Schwann cells by activating the PI3K/AKT signaling pathway. These findings highlight that hUCMSC-EVs is a novel treatment strategy for peripheral nerve injury.

## Data Availability

The data that support the findings of this study are available from the corresponding author upon reasonable request.

## Abbreviations

hUCMSCs: Human umbilical cord mesenchymal stem cells
EVs: Extracellular vesicles
miRNA: MicroRNA
DMEM: Dulbecco's modified Eagle's medium
FBS: Fetal bovine serum
PBS: Phosphate-buffered saline
EP: Eppendorf
CCK-8: Cell counting kit-8
TEM: Transmission electron microscopy
NTA: Nanoparticle tracking assay
DAPI: 4′,6-Diamidino-2-phenylindole
qRT-PCR: Quantitative reverse-transcription polymerase chain reaction
GAPDH: Glyceraldehyde-3-phosphate dehydrogenase
BrdU: 5-Bromo-2′-deoxyuridine
AKT: Protein kinase B
PI3K: Phosphatidylinositol 3 kinase.
